# TRP Channels in Digestive Tract Cancers

**DOI:** 10.3390/ijms21051877

**Published:** 2020-03-09

**Authors:** Paulina Stokłosa, Anna Borgström, Sven Kappel, Christine Peinelt

**Affiliations:** Institute of Biochemistry and Molecular Medicine, National Center of Competence in Research NCCR TransCure, University of Bern, 3012 Bern, Switzerland; anna.borgstroem@ibmm.unibe.ch (A.B.); sven.kappel@ibmm.unibe.ch (S.K.); christine.peinelt@ibmm.unibe.ch (C.P.)

**Keywords:** TRP channel, cancer, gastrointestinal tract, apoptosis, cell cycle, migration, invasion, cancer hallmarks

## Abstract

Cancers of the digestive tract are among the most prevalent types of cancer. These types of cancers are often diagnosed at a late stage, which results in a poor prognosis. Currently, many biomedical studies focus on the role of ion channels, in particular transient receptor potential (TRP) channels, in cancer pathophysiology. TRP channels show mostly non-selective permeability to monovalent and divalent cations. TRP channels are often dysregulated in digestive tract cancers, which can result in alterations of cancer hallmark functions, such as enhanced proliferation, migration, invasion and the inability to induce apoptosis. Therefore, TRP channels could serve as potential diagnostic biomarkers. Moreover, TRP channels are mostly expressed on the cell surface and ion channel targeting drugs do not need to enter the cell, making them attractive candidate drug targets. In this review, we summarize the current knowledge about TRP channels in connection to digestive tract cancers (oral cancer, esophageal cancer, liver cancer, pancreatic cancer, gastric cancer and colorectal cancer) and give an outlook on the potential of TRP channels as cancer biomarkers or therapeutic targets.

## 1. Introduction

Digestive malignancies refer to a heterogeneous group of cancers that arise in the gastrointestinal tract and associated organs. Cancers of the colon and rectum, stomach, liver, esophagus, and pancreas are among five of the ten most prevalent cancers and cancer-related causes of death [1]. For 2020, approximately 387,000 new cases of cancer in the digestive system, the oral cavity and the pharynx and 179,000 related deaths have been estimated in the United States [2]. Successful treatment of these malignancies largely depends on effective screening tools, such as diagnostic blood, stool and endoscopic tests [3,4,5]. Therapeutic approaches include surgery, chemo and radiation therapy, hormone therapy; as well as tailored and personalized therapies, which are presently undergoing development [6,7,8,9,10,11,12,13]. To further advance personalized medicine, many research groups have focused their work on the understanding of the molecular nature of cancer hallmark functions, including unlimited proliferation, increased viability, migration and invasion of cancer cells, and decreased ability to induce apoptosis [14]. Ion channels mediate numerous responses in cellular physiology and are often dysregulated in various diseases, especially in most types of cancer including cancers of the gastrointestinal tract [15]. Ion channels are often responsible for switching on and off intracellular signaling pathways, which makes them attractive candidate drug targets. In addition, they are mostly expressed on the cell surface, and, as a consequence, ion channel targeting drugs do not need to enter the cell. In recent decades, members of the transient receptor potential (TRP) channel family have been proposed as potential biomarkers and/or drug targets in cancer therapy.

TRP genes were first reported in 1969, when a mutation in the genome of a visually impaired *Drosophila melanogaster* fruit fly was described. This mutant, called transient receptor potential, had a transient response to steady light, in opposite to sustained electroretinogram, recorded in the wild-type flies [16]. However, the *trp* gene was identified and described 20 years later [17]. Since then, numerous homologous TRP channel family members have been identified and classified into six human TRP channel subfamilies, including canonical (TRPC), melastatin (TRPM), vanilloid (TRPV), ankyrin (TRPA), polycystic (TRPP), and mucolipin (TRPML) channels [18,19]. Most TRP channels have an essential role in the influx of monovalent and divalent cations, such as Na^+^, Mg^2+^ and Ca^2+^, as well as trace metal ions [18]. Originally, the TRP channel transduction pathways were described for taste and pungent compound perception, thermo- and mechanosensation, pain, osmoregulation, as well as hormone and pheromone signaling [20,21,22,23,24]. Besides their roles in sensory processes, TRP channels mediate many cellular physiological and pathophysiological functions in cancer and the immune system [25,26,27,28,29,30,31,32,33,34]. One way of how TRP channels contribute to the pathogenesis of different types of cancer is through (dys)regulation of intracellular ion levels. For example, the switch from a quiescent cell to a proliferating cell is characterized by global dynamic Ca^2+^ elevations and the activation of Ca^2+^ effectors. Cells progressing through the cell cycle are characterized by Ca^2+^ oscillations [35,36,37,38]. Additionally, Ca^2+^ can contribute to the activation or inhibition of apoptosis, as well as the ability of cells to migrate [35,36,37,38]. All TRP proteins share a common topology of six transmembrane segments (S1–S6), with a pore-forming loop between the S5 and S6 segments. The transmembrane segments tend to share the greatest homology within the particular subfamily, and amino acid sequences in the pore region of TRP channels are the most highly conserved [18,19]. The amino (N) and carboxyl (C) terminuses are located intracellularly; they vary in length and sequence, and contain diverse domains and motifs, which play a role in channel assembly, activation and regulation. These domains and motifs can include coiled coils, calmodulin-binding sites, lipid interaction domains, EF hands, or phosphorylation sites, and are highly variable within members of the same subfamily [18,39]. Recent advances in cryogenic electron microscopy (cryo-EM) based structural analysis have provided insights into the architecture of several TRP channels, including TRPA1, TRPPC, TRPM, and TRPV channels [40,41,42,43,44,45,46,47,48,49,50,51,52,53,54]. To date, most changes involving TRP channels in cancer do not involve mutations in the TRP genes but rather increased or decreased levels of expression of functional TRP proteins, depending on the cancer’s stage. Here, we focus on TRP channels, especially members of the TRPC, TRPM, and TRPV subfamilies in digestive malignancies, that are mostly of epithelial origin, including oral, esophageal, pancreatic, gastric, and colorectal cancer.

## 2. Oral Cancers

Oral squamous cell carcinoma (SCC) accounts for approximately 90% of cases of oral cancer [55]. The oral cavity comes into contact with several sensory stimuli that are known to activate a number of TRP channels. Capsaicin, the chemical compound responsible for the burning sensation of chili peppers, activates TRPV1 channels. Additionally, pungent mustard oil activates TRPA1, and menthol activates TRPM8. It has been suggested that these compounds might exhibit chemoprotective features [56,57,58]. In human oral SCC cells, TRPV1–4, TRPV6, TRPA1, TRPM8, and TRPM2 are expressed [59,60,61,62]. In 2009, the expression levels of TRPV1 were investigated in the human tongue, in tongue SCC, and pre-malignant leukoplakia. Under pre-malignant conditions and in SCC, the TRPV1 protein expression was increased [59]. The expression of TRPV1 protein was also shown in human oral SCC. Capsaicin, a TRPV1 agonist, was shown to induce cytotoxicity in oral SCC cells. However, this effect was independent of TRPV1 conductivity, as these cells did not exhibit an increase in intracellular Ca^2+^ upon stimulation with capsaicin [63]. Furthermore, it was shown that TRPV1–4 expression levels were elevated on protein level in different areas of the oral cavity, including the tongue, buccal mucosa, gingiva, and the oral floor, compared to normal oral mucosa. In addition, known risk factors for SCC, such as alcohol consumption and smoking, increased the expression levels of TRPV1–4 mRNAs [61]. TRPA1 is expressed in oral SCC, and thymol induces an increase in intracellular Ca^2+^, which is blocked when a TRPA1 antagonist is present. However, the anti-cancerous effects of thymol are not mediated by TRPA1 [64]. Notably, it has been reported that oral SCC cells may secrete certain lipids that activate TRPA1 and TRPV1 nociceptors and thus mediate SCC-induced neck pain [65]. Cold/menthol-activated TRPM8 is expressed on protein and mRNA level in oral SCC cell lines. Immunohistochemistry (IHC) analysis showed that TRPM8 localizes not only in the plasma membrane, but also in the endoplasmic reticulum (ER) membrane. Menthol activates TRPM8 in the ER plasma membrane, resulting in a Ca^2+^ release from intracellular Ca^2+^ stores and store-operated Ca^2+^ entry. Menthol-activated Ca^2+^ entry is also partially mediated by TRPM8, and both of these mechanisms can be blocked by a TRPM8-specific inhibitor. Functionally, the inhibition of TRPM8 results in a reduction in oral SCC cell viability, migration and invasion [60]. TRPM2 is another ion channel that contributes to Ca^2+^ influx and is activated by cADPR, reactive oxygen species (ROS), and 2′-deoxy-ADPR [66,67]. TRPM2 protein expression is significantly increased in tongue SCC compared to non-malignant tongue tissues (control or papilloma). In two human tongue SCC cell lines, TRPM2 expression was upregulated compared to a non-tumorigenic oral epithelial cell line. The downregulation of TRPM2 decreased cancer cell migration and increased ROS induced apoptosis [62]. Table 1 summarizes studies in which the expression of TRP channels in human tongue and oral tumor samples was described.

## 3. Esophgeal Cancer

Esophageal cancer is the eighth most common cancer worldwide [1] and is characterized by a poor prognosis [68]. This type of cancer is classified into two different cancer entities: esophageal squamous cell carcinoma (ESCC) and esophageal adenocarcinoma (EAC) [1,68,69,70]. The link between the pathophysiology of esophageal cancer and TRP channel expression has been exclusively made for ESCC and includes dysregulation of TRPC6, TRPM7, TRPM8, TRPV1, TRPV2, and TRPV4 expression levels. Table 2 summarizes studies in which the expression of TRP channels in human ESCC samples was described.

TRPC6 mRNA and protein expression levels are increased in human ESCC tissues compared to normal esophageal tissues. Furthermore, the inhibition of TRPC6 in ESCC cells led to decreased Ca^2+^ signaling and cell cycle arrest via Cdk1. In addition, the inhibition of TRPC6 decreased tumor formation in a mouse xenograft model [71].

TRPM8 mRNA and protein expression have been reported to be upregulated in ESCC tissues and ESCC cell lines. The knockdown and inhibition of TRPM8 decrease proliferation of esophageal cancer cells. Moreover, TRPM8 negatively regulates PD-L1 expression through the calcineurin–NFATc3 pathway, enabling the immune evasion of ESCC cells [72]. 

The protein expression of the Ca^2+^/Mg^2+^ channel TRPM7was reported to be a good independent prognostic factor for patients with ESCC. Additionally, siRNA-based silencing of TRPM7 in TE6 ESCC cells increased their proliferation, migration, and invasion [73].

TRPV2 mRNA and protein have been found to be overexpressed in ESCC cell lines. In ESCC patients, higher expression of TRPV2 protein correlates with a worse 5 year overall survival rate after surgery. The knockdown of TRPV2 in ESCC cells decreased proliferation, cell cycle progression, and the ability to invade and migrate. Moreover, the authors found downregulated WNT/β-catenin or basal cell carcinoma signaling-related genes [74]. TRPV1 and TRPV4 were both found to be expressed on mRNA and protein level in non-tumorous esophageal squamous cells and overexpressed in ESCC cells. Furthermore, TRPV1 and TRPV4 are functional in these cells, as shown with calcium imaging and whole cell patch clamp techniques. Finally, the overactivation of both channels leads to increased proliferation and migration of ESCC cells [75].

## 4. Liver Cancer

Liver can be affected by two types of cancer: hepatocellular carcinoma (HCC) or metastases from colorectal cancer. Liver cancer tissue has been reported to express several TRP channels. El Boustany and colleagues showed that TRPC1, TRPC6, TRPV1, TRPV2, TRPV4, TRPM4, TRPM6, TRPM7, and TRPM8 are expressed on mRNA level in both Huh-7 and HepG2 human hepatoma cell lines, while TRPV3 and TRPM5 are only expressed in Huh-7 cells [76]. Table 3 summarizes studies in which the expression of TRP channels in human liver cancer samples was described.

TRPV1 is overexpressed in hepatocarcinoma tissues compared to normal liver tissue. In the same study, a correlation between TRPV1 protein expression and histopathologic differentiation was reported. High TRPV1 expression was associated with longer disease-free survival [77]. In addition, TRPV1 was previously shown to modulate migration of HCC cells [81,82]. Furthermore, capsaicin induced an increase in intracellular Ca^2+^ levels, ROS production, and apoptosis in HepG2 cells [83]. The induction of apoptosis in HepG2 cells by capsaicin-induced TRPV1 activation was later further confirmed [84]. 

Another member of the TRPV subfamily, TRPV2, is also expressed in hepatocarcinoma tissue. TRPV2 expression on mRNA and protein level are increased in moderately and well-differentiated hepatocarcinoma tissues compared to poorly differentiated tumors. Moreover, a correlation between TRPV2 expression and portal vein invasion was confirmed [78]. Interestingly, the expression of TRPV2 mRNA and protein levels in HepG2 and Huh-7 cells was reported to increase upon exposure to ROS, specifically H_2_O_2_, resulting in an activation of cell death. The elevation of TRPV2 expression led to an inhibition of pro-survival signals (Akt, Nrf2) and, in the early stage of apoptosis, promoted the activation of pro-death signals (p38, JNK1) [85]. Another study showed that shRNA-based TRPV2 knockdown in HepG2 cells enhanced spheroid and colony formation, which was restored by the overexpression of TRPV2. Additionally, the expression of TRPV2 protein was linked to the stemness of liver cancer cells, as the knockdown of TRPV2 in HepG2 cells induced the expression of liver cancer stem-like cells (LCSLCs) markers. Moreover, in human hepatoma cell line SMMC-7721, which exhibits lower TRPV2 protein expression than HepG2, reinforced TRPV2 expression led to the reduction of the expression of LCSLCs markers and reduced spheroid and colony formation. In line with these findings, probenecid, a TRPV2 agonist, also reduced LCSLCs markers expression, as well as spheroid and colony formation. LCSLCs are considered to be good targets for liver cancer therapy, as they exhibit stem cell properties and are of a highly tumorigenic nature. Therefore, TRPV2 was proposed to be a potential target in hepatoma therapy [86]. Notably, reduced expression of TRPV2 mRNA and protein in poorly differentiated tumors in comparison to higher differentiated hepatoma [78] supports the idea that reduced TRPV2 expression promotes the stem-cell features of hepatoma cells during the early stages of tumor development [86]. 

Protein and mRNA levels of TRPV4 are elevated in hepatocellular carcinoma tissue compared with paired non-tumor tissue. Poorly differentiated HCC tumors displayed stronger TRPV4 expression and a positive correlation between TRPV4 expression; the histological grade and number of tumors was described [79]. In addition, with the use of a TRPV4-specific antagonist and agonist, Fang and colleagues showed that TRPV4 is crucial for HCC cell viability and that its inhibition could cause anti-tumor effects through modulation of the expression of apoptosis-related molecules. Furthermore, in a xenograft mouse model system, the blockage of TRPV4 was shown to decrease tumor size and weight compared to the control group [79].

The downregulation of TRPC1 with shRNA-based interference has been shown to suppress cell proliferation but not migration of HCC cells. In addition to the anti-proliferative effect of TRPC1 silencing, Selli and colleagues reported an elevation in store-operated Ca^2+^ entry [87].

TRPC6 is weakly expressed on mRNA and protein level in normal hepatocytes but strongly expressed in liver carcinoma samples. Moreover, high TRPC6 expression has been suggested to be associated with a higher Tumor Node Metastasis (TNM) classification of tumors [76,80]. TRPC6 overexpression in Huh-7 cells leads to increased proliferation [76]. In addition, TGF-β has been shown to induce a formation of the complex between TRPC6 and the type1 Na^+^/Ca^2+^ exchanger (NCX1), leading to an increased migration and invasion of HCC. Moreover, a positive correlation was found between the severity of HCC progression and the expression of TRPC6 and NCX1 [80]. Both the inhibition and RNAi downregulation of TRPC6 or NCX1 was able to attenuate TGF-β induced cell migration or the intrahepatic metastasis of human HCC in an in vivo xenograft mouse model [80]. Moreover, Wen and colleagues recently reported a significantly increased TRPC6 mRNA expression in HCC cells upon hypoxia stimulation, doxorubicin treatment, or ionizing radiation [88]. The inhibition or downregulation of TRPC6 significantly decreased drug resistance to all three stimuli, and TRPC6 inhibition could reverse endothelial–mesenchymal transition (EMT), induced by doxorubicin treatment. In line with these findings, TRPC6 interference in vivo enhanced the sensitivity to doxorubicin and led to slower growth of the cells compared to the control cells [88]. 

Another study found that bradykinin can activate TRPM7 in HepG2 cells, leading to an influx of Ca^2+^. TRPM7-mediated Ca^2+^ influx was necessary for the activity of calpains, which play a role in migration of HepG2 cells. These findings suggest that TRPM7 could be involved in migration of liver cancer cells. Notably, treatment with bradykinin resulted in an increase in MMP-2 secretion and enhanced migration [89]. 

## 5. Pancreatic Cancer

Pancreatic cancer is usually detected at an advanced stage and, currently, most patients are diagnosed with distant metastases and, therefore, have a poor 5 year survival rate [90,91]. Detection of pancreatic cancer at an early stage has a favorable impact on long-term survival, as the 5 year survival of localized pancreatic cancer is about 25% but is only 2% for distant disease [90,91]. 

An analysis performed by Lin and colleagues revealed that somatic mutations in the *TRPM2* gene exhibit a negative correlation with pancreatic cancer patient survival rates in comparison to the group without *TRPM2* mutations. Additionally, the higher the TRPM2 mRNA expression in the cancerous tissue, the shorter the survival of the patients. TRPM2 expression was also shown to enhance proliferation, migration, and invasion of PANC-1 cells, a human pancreatic cancer cell line [92]. 

In 2007, it was shown that TRPM8 is expressed on mRNA and protein level and mediates non-selective cation currents in the human pancreatic neuroendocrine tumor (NET) cell line BON, as well as in primary NET cell lines [93]. TRPM8 protein was found to be upregulated in human pancreatic adenocarcinoma cell lines and tissues [94,95]. Other studies further confirmed the upregulation of TRPM8 in pancreatic cancer tissues in human patients compared to adjacent tissues [96,97]. In addition, high TRPM8 protein expression was found to be associated with lower overall survival and poor disease free survival values for pancreatic cancer patients [97], as well as positively correlated with the tumor size and stage of pancreatic cancer [94]. Furthermore, it was shown that TRPM8 is required for proliferation of the pancreatic adenocarcinoma cell lines, PANC-1 and BxPC-3, due to the cell cycle arrest in the G0/G1 phase [95]. Another study showed that TRPM8 protein is expressed in PANC-1 cells in a non-glycosylated form and is functional. Additionally, TRPM8 knockdown with siRNA resulted in a decrease in cell migration [98]. TRPM8 expression in a non-glycosylated form was further confirmed and shown to have an impact on the conductive properties of the channel. Additionally, it was suggested that the glycosylation patterns in PANC-1 cells could have an impact on proliferation [99]. It was also further shown that TRPM8 mediates Ca^2+^ influx in PANC-1 and BxPC-3 cell lines and plays a role in proliferation as well as migration. Additionally, targeting TRPM8 in PANC-1 and BxPC-3 with siRNA resulted in an enhancement of gemcitabine cytotoxicity, which was accompanied by a change in the expression of apoptosis- and gemcitabine metabolism-related proteins [96]. Another study further showed that targeting TRPM8 with shRNA in BxPC-3 and MIA PaCa-2 resulted in a decrease in invasion [94]. 

TRPM7 was shown to play a role in the development of the pancreas in a zebra fish model, which was linked to Mg^2+^ sensitive signaling. These findings could be translated to the pathogenesis of pancreatic adenocarcinoma, in which TRPM7 protein was shown to be overexpressed and necessary for Mg^2+^-regulated proliferation. Targeting TRPM7 with siRNA in PANC-1 and BxPC-3 resulted in a cell cycle arrest in the G0/G1 phase, accompanied by a change in the expression of p21, cyclin G1, and cyclin B1. The role of TRPM7 in the regulation of pancreatic cancer cells proliferation was also connected to Mg^2+^, as supplementation of the culture medium with Mg^2+^ reversed the decrease in proliferation caused by the knockdown of TRPM7 [100]. TRPM7 does not affect apoptosis in PANC-1 and BxPC-3 [100,101]. However, it plays a role in preventing replicative senescence. Targeting TRPM7 enhances cytotoxicity of the conventionally used chemotherapeutic, gemcitabine, which is a pro-apoptotic agent, suggesting that targeting TRPM7 in pancreatic cancer could support standard chemotherapy [101]. Another group showed that TRPM7 mRNA and protein are overexpressed in pancreatic ductal adenocarcinoma compared to healthy pancreatic tissue, and TRPM7 staining intensity is inversely correlated with patients’ survival. Additionally, the study showed that BxPC-3 cells exhibit TRPM7-characteristic non-selective cationic currents and that TRPM7 activity regulates intracellular Mg^2+^ levels [102]. Contrary to previous findings [100,101], another study based on the siRNA targeting of TRPM7 reported no effect on cell viability and proliferation but showed a decrease in cell migration [102]. The overexpression of TRPM7 protein in pancreatic cancer was further confirmed, and a correlation was found between TRPM7 expression levels and the size and stage of tumors. Additionally, TRPM7 is overexpressed in pre-malignant tissue. Therefore, the expression of TRPM7 could be further investigated as a potential biomarker [103]. Furthermore, high TRPM7 staining in pancreatic ductal adenocarcinoma has been associated with higher TRPM7 protein staining in the lymph nodes, suggesting that TRPM7 might be involved in invasion of pancreatic cancer cells [104]. Indeed, TRPM7 has been shown to be involved in the invasion of BxPC-3 cells [103]. Another study showed that in PANC-1 and MIA PaCa-2 cell lines, the TRPM7-mediated cation current regulates Mg^2+^ homeostasis and cell invasion, as TRPM7 knockdown was shown to reduce invasion along with a decrease in MMP2, uPA, and Hsp90α secretion [104]. 

TRPV1 was shown to be expressed on mRNA and protein level and regulate intracellular Ca^2+^ levels in pancreatic NET BON-1 cells, and its activity triggered secretion of chromogranin A [105]. Furthermore, the TRPV1 agonist, capsaicin, reduced viability and proliferation, and induced apoptosis in NET cells. The cytotoxicity of capsaicin was shown to be due to the disturbance of mitochondrial potential and the inhibition of ATP production [106]. Similar to the observations in NET cells, TRPV1 overexpression in PANC-1 cells inhibited cell proliferation. Either the overexpression or agonist-induced activation of TRPV1 reduced the expression of epidermal growth factor receptor (EGFR), due to its ubiquitination. Additionally, TRPV1 reduced mRNA expression of two oncogenes, KRAS and AKT2 [107]. Another member of the TRPV subfamily, TRPV6, is expressed in BON-1 cell line on mRNA and protein level, and it regulates Ca^2+^ levels. The downregulation of TRPV6 in BON-1 cells, resulted in an inhibition of proliferation and reduced NFAT activation [108]. Furthermore, TRPV6 protein expression is elevated in pancreatic cancer tumors compared to non-tumor adjacent tissues and its high expression correlated with lower survival of the patients. The knockdown of TRPV6 in pancreatic cancer cell lines resulted in a reduced proliferation, induced cell cycle arrest in the G0/G1 phase, reduced migration and invasion, as well as increased apoptosis [109]. 

Table 4. summarizes studies in which the expression of TRP channels in human pancreatic cancer samples was described.

## 6. Gastric Cancer

Gastric cancer was the third most common cause of cancer-related deaths in 2018, just after lung and colorectal cancer [1]. Most patients with early-stage gastric cancer are asymptomatic; therefore, a diagnosis is often made when the cancer is at an advanced stage and shows metastasis [110]. Several TRP channels have been proposed to be involved in the pathogenesis of gastric cancer. The TRPC6 channel was shown to be upregulated on protein and mRNA level in human gastric cancer epithelial cells in comparison to normal gastric epithelial cells. TRPC6-mediated Ca^2+^ influx in gastric cancer cell lines is responsible for regulation of the cell cycle, as the inhibition of TRPC6 resulted in cell cycle arrest in the G2/M phase and inhibited cell growth. The involvement of TRPC6 conductivity in cell cycle regulation was further confirmed in experiments where the TRPC6 dominant-negative mutant was expressed. Moreover, treatment of nude mice with a TRPC6 blocker resulted in the inhibition of the development of a xenografted human gastric tumor [111]. Another study suggested a role for TRPC 1/3/6 in the regulation of EMT [112]. Additionally, a newly developed TRPC6 inhibitor showed an anti-tumor effect in nude mice with a xenografted human gastric tumor [113]. 

Capsaicin has been shown to induce apoptosis in a normal human epithelial gastric cell line and the gastric cancer cell line AGS. However, AGS cells were found to be more susceptible to capsaicin-induced apoptosis, which was induced through an increase in mitochondrial permeability and activation of Bax and p53. Surprisingly, the capsaicin-induced apoptosis was dependent on Ca^2+^ influx mediated by TRPV6, rather than TRPV1, a known capsaicin receptor [114]. 

TRPM2 was found to be expressed on mRNA level in gastric cancer patients, and its high expression was negatively associated with the overall survival of patients. Functional TRPM2 is expressed in gastric cancer cell lines AGS and MKN-45, and its shRNA based knockdown results in the inhibition of proliferation and enhancement of apoptosis. Additionally, TRPM2 knockdown was shown to alter autophagy in AGS cells, which led to mitochondrial dysfunction. The knockdown of TRPM2 also sensitized AGS and MKN-45 cells to treatment with paclitaxel and doxorubicin, resulting in a further reduction in cell viability. These findings suggest that targeting TRPM2 in combination with standard chemotherapeutic drugs could be beneficial for the treatment of gastric cancer patients [115]. 

TRPM7 channel was shown to be expressed in gastric cancer cell lines on mRNA and protein level. AGS gastric cancer cells exhibit TRPM7-like currents, and suppression of these currents by the unspecific TRPM7 inhibitors, La^3+^ or 2-APB, resulted in a decrease in cell viability and higher apoptosis rates. Additionally, Mg^2+^ was necessary for AGS cells survival and growth [116]. 

Table 5 summarizes studies in which the expression of TRP channels in human gastric cancer samples was described. 

## 7. Colorectal Cancer

Colorectal cancer (CRC) is one of the most frequent types of cancer and cancer-related cause of death, both in Europe and in the United States [117,118]. Several TRP channels have been shown to be dysregulated in CRC. A study investigating mRNA expression levels of the TRP channels in human CRC tissue versus normal colon mucosa detected an increase in gene expression of TRPM8, TRPV6, and TRPV1 and a lower expression of TRPV4, TRPM4, TRPV3, TRPC6, and TRPV5 in the tumor tissues of CRC compared to normal tissues [119]. Table 6 summarizes studies in which the expression of TRP channels in human gastric cancer samples was described.

TRPC1 ion channel, which displays permeability towards Ca^2+^ ions, was reported to be upregulated on mRNA and protein level in CRC cells [124], and its higher mRNA expression in CRC patients has been correlated with a poor prognosis [120]. The upregulated expression of TRPC1 in CRC has been shown to contribute to an increased Ca^2+^ influx via store operated Ca^2+^ entry and higher Ca^2+^ signaling, which resulted in an increased proliferation, invasion, and survival of CRC cells [124]. Furthermore, Ca^2+^ influx through TRPC1 was also linked to an increased migration of CRC cells [125]. Another member of the TRPC subfamily, which conducts Ca^2+^, TRPC5, was shown to play a role in the drug resistance of human CRC cell lines. HCT-8 and LoVo cells resistant to 5-fluorouracil (5-FU), which is a commonly used chemotherapeutic in CRC therapy, showed higher expression of TRPC5 mRNA and protein in comparison to non-resistant cells. Further investigations revealed that the TRPC5-mediated Ca^2+^ influx induces the expression of the ATP-binding cassette subfamily B member 1 (ABCB1), a pump overproduced in cancer cells, responsible for the export of cytotoxic drugs. Additionally, TRPC5 promoted nuclear β-catenin localization [126].

Increasingly, evidence suggests an important role for TRPV1 channel in the physiology and pathophysiology of the intestine. Increased expression of TRPV1 protein was found in the colon of patients with inflammatory bowel disease, which was linked to an increased pain sensation [127]. However, several studies suggested that the expression and activity of TRPV1 in the intestine has a protective role in inflammatory states [128,129,130,131]. Consistent with these findings, in a mice model of colitis-associated cancer (CAC), mice that lacked the expression of TRPV1 had a higher incidence and higher number of tumors in the distant colon. The lack of TRPV1 was also accompanied by an increased proliferation of colon cells and higher β-catenin localization to the nuclei. Additionally, tumors from mice lacking TRPV1 showed an increased infiltration of inflammatory cells into the tumors, along with an elevated expression of IL-6 and IL-11 and activation of STAT3 and NF-kB signaling pathways [132]. Further evidence shows that TRPV1 protein expression can have a protective role against tumor development. De Jong and colleagues showed that functional TRPV1 is expressed in intestinal epithelial cells and can be activated by capsaicin. TRPV1 activation inhibits EGFR-induced epithelial cell proliferation via activation of Ca^2+^/calpain. In a murine model of multiple intestinal neoplasia (Apc^Min/+^ mice), TRPV1 deficiency promoted intestinal adenoma formation correlated with a reduced lifespan [133], which was consistent with previous findings [132]. In this model, mice that lack TRPV1 showed higher EGFR phosphorylation and proliferation markers in intestinal epithelial cells, and the deletion of TRPV1 increased the expression of the EGFR-regulated oncogenes, *c-Fos* and *c-Myc*. Furthermore, the administration of dietary capsaicin increased the survival of Apc^Min/+^ mice in a process that was dependent on TRPV1 [133]. Another study suggested that TRPV1 protein expression is decreased in CRC tissues. Treatment of HCT116 cell line with capsaicin resulted in an inhibition of proliferation and induced apoptosis through the activation of the tumor suppressor, p53. In this cell line, treatment with capsaicin led to an increase in intracellular Ca^2+^, possibly through TRPV1 [121]. On the other hand, studies in human CRC cell lines showed that Fibrulin-5, a component of ECM, is downregulated in CRC tissues and cell lines. Fibrulin-5 induces apoptosis through the downregulation of TRPV1 and ROS production [134]. Another study in HT-29 cell line showed that capsaicin induced apoptosis through PPARγ signaling but without the involvement of TRPV1, since capsazepine, a specific antagonist for the vanilloid receptor, did not inhibit capsaicin induced apoptosis [135]. 

TRPM8 is another ion channel responsible for Ca^2+^ influx into cells and was found to be highly expressed on protein level in human CRC cell lines, Caco-2, and HCT116. Furthermore, cannabigerol, a non-psychotropic cannabis-derived cannabinoid, reduces colon cancer progression in vivo and selectively inhibits the growth of CRC cells via interaction with TRPM8 [136].

TRPM4 is an ion channel, which is directly activated by an increase in intracellular Ca^2+^ concentration, however, is not permeable towards Ca^2+^. In non-excitable cells, under physiological conditions, TRPM4 conducts Na^+^ into the cell, thereby contributing to plasma membrane depolarization. This, in turn, reduces the driving force for further Ca^2+^ entry through store operated Ca^2+^ channels. Therefore, TRPM4 is regarded to be a negative regulator of Ca^2+^ signaling [137,138,139,140]. Reports on mRNA expression levels of TRPM4 in colorectal cancer either found it to be lower in CRC tissue compared to normal colon tissue [119], or no differences were detected [141]. However, a recent study investigating TRPM4 protein expression levels in CRC tissues showed that its high expression correlates with high numbers of tumor buds and an increased percentage of infiltrative tumor border configuration. Both of these features correlate with an increased frequency of vessel invasion and lymph node metastasis, which ultimately lead to an increased probability of disease reoccurrence and cancer related death. TRPM4 protein was also shown to be highly expressed in tumor buds, which were linked to increased metastasis in CRC. Furthermore, TRPM4-mediated Na^+^ influx was shown to regulate cell viability and the cell cycle of HCT116 cells. In the same study, TRPM4 was also linked to the regulation of CRC cells migration and invasion [122]. 

There are two Mg^2+^ channels in the superfamily of TRP channels, TRPM6 and TRPM7, and their activity is linked to the pathogenesis of CRC. TRPM6 and TRPM7 are unique ion channels that mediate Mg^2+^ homeostasis, as well as proteins, combining an ion channel with a functional α-kinase domain [29,142,143,144,145]. The role of Mg^2+^ in the pathogenesis of CRC has been reported. Higher Mg^2+^ intake seems to be associated with a modest reduction in the risk of CRC, in particular, colon cancer [146]. However, studies showed that mice receiving Mg^2+^-deficient diet had a significant retardation of their primary tumor growth [147]. Therefore, the relationship between Mg^2+^ intake and cancer might be more complex, especially since the relationship of Ca^2+^:Mg^2+^ intake is important for the regulation of cellular responses [148]. At the cellular level, Mg^2+^ can exhibit either anti- or pro-tumor effects and has been suggested to contribute to different mechanisms involved in carcinogenesis, such as cancer cell proliferation, metabolic reprogramming, the ability to metastasize, and neo-angiogenesis [149,150]. Mg^2+^ was shown to contribute to the regulation of cell proliferation and cell cycle, and Mg^2+^ deficiency has been shown to induce cell cycle arrest in the G0/G1 phase [148]. On these bases, TRPM6 and TRPM7 could be potential players in CRC. Indeed, it was reported that TRPM6 is downregulated in CRC tissues on mRNA level [120,123], and its higher expression is correlated with an increased overall survival [123]. On the other hand, TRPM7 was suggested to be upregulated in CRC on mRNA level [151]. Moreover, a single-nucleotide polymorphism that substitutes TRPM7 threonine 1482 for isoleucine (T1482I) increases the risk of the development of colon cancer, particularly in patients with a high Ca^2+^/Mg^2+^ ratio [152]. It must be noted that this mutation does not influence kinase activity, nor the channel’s conductivity, but the channel’s sensitivity to Mg^2+^ [153]. TRPM7 was also shown to be overexpressed in CRC cell lines. The downregulation of TRPM7 suppressed CRC cell proliferation, migration, and invasion, as well as triggered cell cycle arrest in the G0/G1 phase, reduced the S phase, and promoted apoptosis. Furthermore, the decrease in TRPM7 expression in CRC cells reversed EMT, which was accompanied by a downregulation in N-cadherin and an upregulation of E-cadherin expression [151]. Mg^2+^ homeostasis regulation via TRPM6 and TRPM7 was also linked to the sensitivity of CRC cells to doxorubicin, a common chemotherapeutic agent. A study in the CRC cell line LoVo showed that cells resistant to doxorubicin have increased total intracellular Mg^2+^ levels compared to sensitive LoVo cells. However, mRNA expression of TRPM6 in cells resistant to doxorubicin was downregulated compared to cells sensitive to doxorubicin. Further, in LoVo cells resistant to doxorubicin, TRPM7 is downregulated on protein level, but not on mRNA level, compared to doxorubicin-sensitive LoVo. Therefore, the levels of TRPM6 and TRPM7 are inversely related to the amounts of total intracellular Mg^2+^. This could be partially explained by the fact that TRPM7 was markedly reduced in resistant cells because of the activation of calpains, which are dependent on intracellular Ca^2+^ levels. Free Ca^2+^ levels are higher in cells resistant to doxorubicin [154]. These findings further support the idea that pathogenesis of CRC might be dependent on the Ca^2+^/Mg^2+^ ratio [152] and suggest a potential interplay between Ca^2+^ and Mg^2+^ transport (dys-) regulation in CRC. Nevertheless, TRPM7 is involved in the modulation of drug resistance in LoVo cells, as downregulation of TRPM7, but not TRPM6, expression enhances viability of LoVo cells exposed to doxorubicin [154]. 

## 8. Conclusions and Outlook

**Figure 1:**
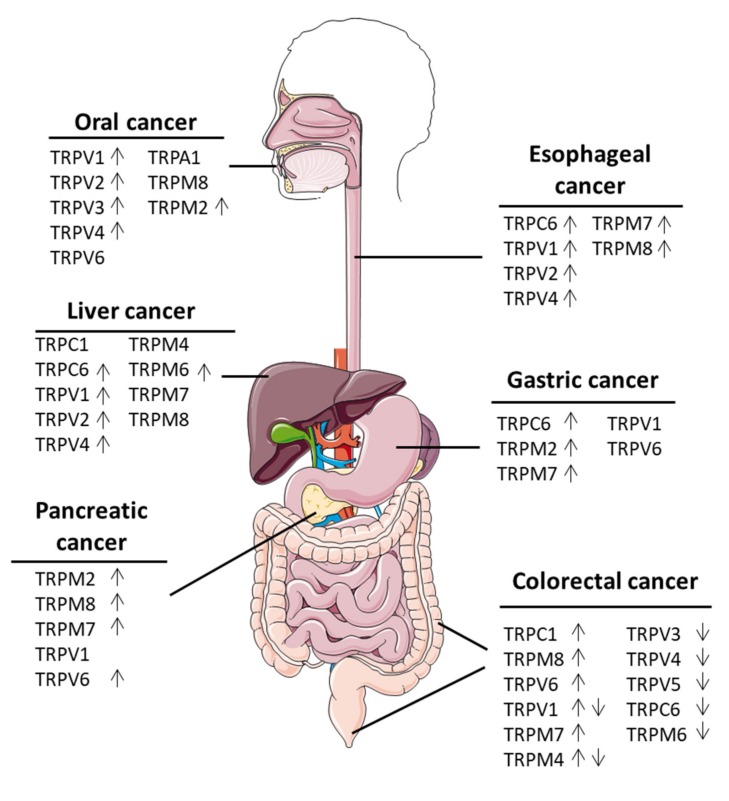
Expression of TRP channels in gastrointestinal tract. Overview of TRP channels expressed in cancers of the gastrointestinal tract or in the cell lines originating from these types of cancers. ↑Arrows indicates that the channel is upregulated, while ↓ arrows indicates that the channel is downregulated. For some channels, there was more than one study, showing that they are either upregulated or downregulated, which is indicated by ↑↓.

Recent studies show that digestive malignancies are characterized by different expression patterns of TRP channels (Figure 1). In general, cancers of the gastrointestinal tract are characterized by a poor prognosis, largely due to late diagnosis, when the disease is at an advanced stage. Therefore, there is a need to develop novel biomarkers for the detection of early stage diseases. Whether TRP channels could play such a role remains under investigation, as recent evidence is limited. For example, TRPM7 is upregulated in pre-malignant pancreatic cancer compared to normal pancreatic tissue [103], and so it could potentially serve as a candidate for a pancreatic cancer biomarker. Another candidate for a novel biomarker could be TRPM4, which was shown to be highly expressed in tumor buds in CRC cancer tissues [122]. Currently, there is a need for the introduction of novel additional biomarkers, which could support the TNM staging system [155]. Tumor budding has been suggested to be an additional prognostic factor, as it is strongly predictive of lymph node metastases, recurrence, and cancer-related death at 5 years [156,157]. Therefore, the expression of TRPM4 could potentially add to the detection of tumor buds. 

**Figure 2:**
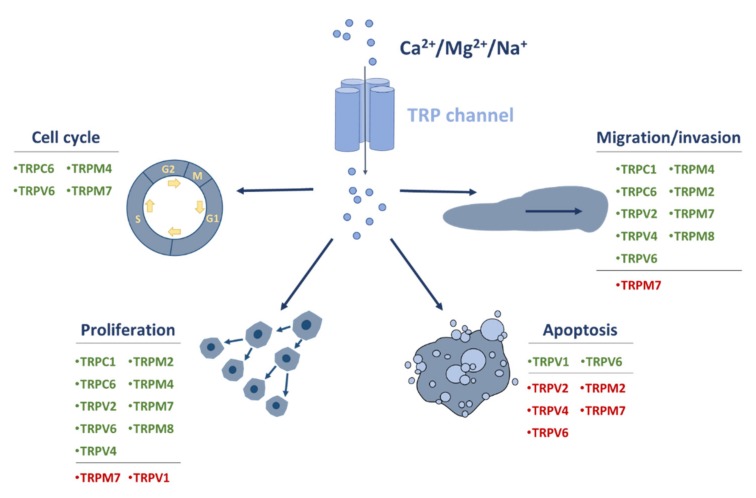
Overview of the role of TRP channels in cell functions of cancers of the gastrointestinal tract. TRP channels, which are permeable to monovalent and divalent cations, such as Na^+^, Ca^2+^ and Mg^2+^, are often dysregulated in cancer cells. These can lead to enhancement/suppression of proliferation, migration and invasion, cell cycle progression, and apoptosis. Channels with activity promoting a particular function are marked in green. Channels with activity suppressing a particular function are marked in red.

Moreover, TRP channels could be potential candidates for therapeutic targets, especially since they are usually expressed on the cell surface, which makes them accessible to small-molecule inhibitors and biological molecules, such as monoclonal antibodies and fusion proteins [158,159,160]. Given the fact that some TRP channels are ubiquitously expressed, the delivery of TRP channel inhibitors to the tumor sites could be supported by cancer cell-specific drug delivery systems that are currently being developed [161]. TRP channels were shown to regulate cellular responses associated with tumorigenesis (summary in Figure 2). A number of studies show the anti-tumor properties of capsaicin, a TRPV1 channel agonist [106,114,121,133,135]. These findings suggest that the activation of TRPV1 could potentially enhance standard therapies. Furthermore, targeting other TRP channels showed an amplification of the effects of commonly used chemotherapeutics [77,96,101,115,126,154], potentially providing an option for the enhancement of current therapeutic strategies. Currently, several clinical trials evaluating ion channel inhibitors and blockers are ongoing [158,162,163]. Furthermore, phase 1 clinical studies evaluating TRPV6 channel inhibitor, SOR-C13, in patients with advanced solid epithelial tumors are being evaluated (NCT01578564 and NCT03784677). While the second study is in the recruiting stage, the results of the first study show that that SOR-C13 was well tolerated. Moreover, of 22 evaluable patients, 54.5% showed stable disease ranging from 2.8 to 12.5 months, which suggests an anti-tumor activity of SOR-C13 [164]. This is a proof of principle, showing that, in the future, drugs targeting TRP channels in cancer could enter clinical use. Future studies will further investigate the potential of TRP channels as therapeutic targets in cancer. 

## Figures and Tables

**Table 1:** Expression of transient receptor potential (TRP) channels in human oral cancers.

Type of Cancer	Channel	mRNA/Protein (+ Assessment Method)	Sample Size	Aim/Outcome + Reference
Oral	TRPV1–4	mRNA (qPCR)	37 oral SCC + tissues samples/compared to normal adjacent tissue	TRPV1–4 mRNA and protein expression upregulated in oral SCC tissue samples in comparison to normal tissue [61]
		protein (IHC)		
	TRPV1	Protein (IHC + WB)	18 tongue SCC + 8 leukoplakia + 7 normal tongue tissues samples	TRPV1 protein and mRNA expression upregulated in tongue SCC tissue samples in comparison to normal tissue [59] ^1^
		mRNA (qPCR)		
		Protein (IHC)	3 oral SCC + 3 normal oral mucosa tissue samples	TRPV1 protein is expressed in oral SCC [63]
	TRPM2	Protein (IHC)	9 normal tongue + 12 papilloma tongue + 23 tongue carcinoma tissue samples	TRPM2 protein is overexpressed in tongue carcinoma in comparison to normal and papilloma samples [62]

^1^ Only IHC assessment of protein expression showed upregulation of TRPV1 in leukoplakia in comparison to normal tissue; data not available for qPCR and WB. IHC, immunohistochemistry; SCC, squamous cell carcinoma; qPCR, quantitative polymerase chain reaction; WB, Western-Blot.

**Table 2:** Expression of TRP channels in human esophageal squamous cell carcinoma (ESCC).

Type of Cancer	Channel	mRNA/Protein (+ Assessment Method)	Sample Size	Aim/Outcome + Reference
Esophageal	TRPC6	mRNA (in situ hybridization)	55 paraffin-embedded ESSC + 21 fresh ESCC tissue samples/compared to normal adjacent tissue	TRPC6 mRNA and protein are overexpressed compared to normal adjacent tissue [71]
		Protein (IHC)		
	TRPM8	mRNA (qPCR)	10 ESCC tissue samples/compared to normal adjacent tissue	TRPM8 mRNA and protein are overexpressed compared to normal adjacent tissue [72]
		Protein (WB)		
	TRPM7	Protein (IHC)	52 ESCC tissue samples/compared to non-cancerous esophageal epithelia	TRPM7 protein is overexpressed compared to non-cancerous esophageal epithelia (no TRPM7 expression detected) [73] ^1^
	TRPV2	Protein (IHC)	62 ESCC tissue samples	Analysis of TRPV2 expression (low/high); worse overall survival and 5 year survival of patients with high TRPV2 protein expression [74]

^1^ The 5 year survival rate of patients with high TRPM7 expression (82.6%) was significantly higher than that of the patients with low expression (54.6%). ESCC, esophageal squamous cell carcinoma; IHC, immunohistochemistry; qPCR, quantitative polymerase chain reaction; WB, Western-Blot.

**Table 3:** Expression of TRP channels in human liver cancer samples.

Type of Cancer	Channel	mRNA/Protein (+ Assessment Method)	Sample Size	Aim/Outcome + Reference
Liver	TRPV1	mRNA (RT-PCR)	6 pairs of HCC tissue samples/compared to normal adjacent tissue	6 non-tumor tissues showed TRPV1 mRNA overexpression; HCC tissue samples showed downregulation in 4/6 tested [77]
		mRNA (in situ hybridization)	15 HCC samples/compared to normal adjacent tissue	TRPV1 expressed in some HCC and normal tissue samples; data non-conclusive [77]
		Protein (IHC)	62 HCC tissue samples + 62 non-tumor control tissues	High TRPV1 expression was observed in 30/62 HCC samples; high TRPV1 expression was associated with longer disease-free survival [77]
	TRPV2	Protein (IHC)	55 HCC cancer tissue samples	Upregulation of TRPV2 on mRNA and protein levels inversely correlated with histopathologic differentiation [78]
		mRNA (RT-PCR)	13 paired HCC tumor mRNA extracts	
	TRPV4	Protein (IHC)	45 HCC tissue samples/compared to normal adjacent tissue	TRPV4 protein and mRNA levels higher in HCC tissues than in normal tissues; positive correlation between TRPV4 expression; the histological grade and number of tumors [79]
		mRNA (qPCR)		
	TRPC6	Protein (IHC)	150 HCC tissue samples/compared to normal tissues	TRPC protein upregulated in HCC tissues in comparison to normal tissues [80]

IHC, immunohistochemistry; qPCR, quantitative polymerase chain reaction; RT-PCR, reverse transcription polymerase chain reaction; WB, Western-Blot.

**Table 4:** Expression of TRP channels in human pancreatic cancer samples.

Type of Cancer	Channel	mRNA/Protein (+ Assessment Method)	Sample Size	Aim/Outcome + Reference
Pancreatic	TRPM2	mRNA (analysis of previously published cancer genome studies)	91 pancreatic cancer patients	High TRPM2 expression correlated with lower overall survival [92]
	TRPM8	Protein (IHC)	280 pancreatic adenocarcinoma tissue microarrays	Moderate or high level of TRPM8 protein expression in 92% of pancreatic adenocarcinoma; the expression levels of TRPM8 positively correlate with the size of the primary tumor and tumor stages [94]
		Protein (IHC)	5 pancreatic adenocarcinoma tissue samples/compared to normal adjacent tissue	TRPM8 protein expression upregulated compared to normal tissue [95]
		Protein (IHC) mRNA (qPCR)	44 pancreatic adenocarcinoma tissue samples/compared to normal adjacent tissue	TRPM8 protein and mRNA upregulated compared to normal tissue [96]
		mRNA (qPCR)	110 pancreatic adenocarcinoma tissue samples/compared to normal adjacent tissue	TRPM8 mRNA upregulated compared to normal tissue; high TRPM8 protein expression was found to be associated with lower overall survival and poor disease free survival values for pancreatic cancer patients [97]
	TRPM7	Protein (IHC)	5 pancreatic adenocarcinoma tissue samples/compared to normal pancreatic tissue samples	TRPM7 protein upregulated compared to normal tissue [100]
		Protein (IHC)	282 pancreatic adenocarcinoma tissue microarrays/compared to normal pancreatic tissue microarrays	TRPM7 protein upregulated compared to normal tissue; TRPM7 expression correlates with the tumor stage [103]
		Protein (IHC) mRNA (RT-PCR)	8 tumor pancreatic ductal adenocarcinoma/compared to 6 normal pancreatic tissues	TRPM7 protein and mRNA upregulated compared to normal pancreatic tissue [102]
	TRPV6	Protein (IHC)	76 tumor pancreatic tissue samples compared to adjacent normal pancreatic tissues	TRPV6 protein upregulated compared to normal pancreatic tissue [109]

IHC, immunohistochemistry; qPCR, quantitative polymerase chain reaction; RT-PCR, reverse transcription polymerase chain reaction.

**Table 5:** Expression of TRP channels in human gastric cancer samples.

Type of Cancer	Channel	mRNA/Protein (+ Assessment method)	Sample Size	Aim/Outcome + Reference
Gastric	TRPC6	Protein (IHC)	25 primary gastric cancer samples/compared to 4 gastritis samples	TRPC6 mRNA and protein expression upregulated compared to gastritis samples [111]
		mRNA (in situ hybridization)	10 primary gastric cancer samples	
	TRPM2	mRNA (analysis of online gastric cancer databases)	896 gastric cancer patients; analysis of low TRPM2 vs high TRPM2 expression	High TRPM2 mRNA expression high expression negatively associated with the overall survival of patients [115]

IHC, immunohistochemistry.

**Table 6:** Expression of TRP channels in human colorectal cancer (CRC) samples.

Type of Cancer	Channel	mRNA/Protein (+ Assessment Method)	Sample Size	Aim/Outcome + Reference
Colorectal (CRC)	TRPC1	mRNA (analysis of CRC datasets, available from public databases)	656 CRC samples including 47 normal samples	High TRPC1 expression correlated with poor prognosis for the patients [120]
			585 CRC samples including 19 normal samples	
	TRPV1	Protein (IHC)	10 CRC tissue samples, 10 CRC-adjacent tissue samples, and 6 normal subjects	TRPV1 protein expression decreased in CRC tissues compared to normal tissues [121]
	TRPM4	Protein (IHC)	CRC tumor tissue microarrays from 379 patients	High TRPM4 protein expression was associated with unfavorable tumor features characteristic for epithelial-mesenchymal transition and infiltrative growth patterns [122]
	TRPM6	mRNA (analysis of CRC datasets, available from public databases)	656 CRC samples including 47 normal samples	TRPM6 mRNA expression decreased compared to normal tissue [120]
			585 CRC samples including 19 normal samples	
		mRNA (analysis of CRC dataset, available from public databases)	585 CRC samples including 19 normal samples	TRPM6 mRNA expression decreased compared to normal tissue; high TRPM6 mRNA expression positively correlated with overall survival [123].

CRC, colorectal cancer; IHC, immunohistochemistry.

## Abbreviations

5-FU	5-Fluorouracil
ABCB1	ATP-Binding Cassette Subfamily B Member 1
Ca^2+^	Calcium Ions
CAC	Colitis-Associated Cancer
CRC	Colorectal Cancer
Cryo-EM	Cryogenic Electron Microscopy
EAC	Esophageal Adenocarcinoma
EGFR	Epidermal Growth Factor Receptor
EMT	Endothelial–Mesenchymal Transition
ER	Endoplasmic Reticulum
ESCC	Esophageal Squamous Cell Carcinoma
HCC	Hepatocellular Carcinoma
IHC	Immunohistochemistry
La^3+^	Lanthanum Ions
LCSLCs	Liver Cancer Stem-Like Cells
Mg^2+^	Magnesium Ion
Na^+^	Sodium Ions
NET	Neuroendocrine Tumor
ROS	Reactive Oxygen Species
SCC	Squamus Cell Carcinoma
TNM	Tumor Node Metastasis
TRP	Transient Receptor Potential
TRPA	Transient Receptor Potential Ankyrin
TRPC	Transient Receptor Potential Canonical
TRPM	Transient Receptor Potential Melastatin
TRPML	Transient Receptor Potential Mucolipin
TRPP	Transient Receptor Potential Polycystic
TRPV	Transient Receptor Potential Vanilloid
WB	Western-Blot
